# SUBJECT INDEX TO VOLUME 6 (2019)

**DOI:** 10.1093/nsr/nwaa013

**Published:** 2020-02-04

## SPECIAL TOPICS: Quantum Computing

### GUEST EDITORIAL

**Table:** 

**20 (1)**	**Preface to special topic on quantum computing**
	Guang-Can Guo and Mingsheng Ying

### RESEARCH HIGHLIGHT

**Table:** 

**21 (1)**	**The complexity-theoretic Bell inequality**
	Zhengfeng Ji

### PERSPECTIVES

**Table:** 

**22 (1)**	**Quantum supremacy: some fundamental concepts**
	Man-Hong Yung
**24 (1)**	**Quantum computing with neutral atoms**
	Mark Saffman
**26 (1)**	**Quantum machine learning**
	Jonathan Allcock and Shengyu Zhang
**28 (1)**	**Model-checking quantum systems**
	Mingsheng Ying and Yuan Feng

### REVIEW

**Table:** 

**32 (1)**	**Semiconductor quantum computation**
	Xin Zhang, Hai-Ou Li, Gang Cao, Ming Xiao, Guang-Can Guo and Guo-Ping Guo

## Topological Matter

### GUEST EDITORIAL

**Table:** 

**195 (2)**	**Preface: special topic on topological matter**
	Fu-Chun Zhang

### PERSPECTIVES

**Table:** 

**196 (2)**	**Majorana gets an iron twist**
	Lingyuan Kong and Hong Ding
**197 (2)**	**Probe Majorana zero modes through their spins**
	Yi Zhou
**199 (2)**	**Superconductivity in topological semimetals**
	Jian Wang
**202 (2)**	**Quantum anomalous Hall heterostructures**
	Ke He and Qi-Kun Xue
**204 (2)**	**Automatic search for topological materials shifts paradigm**
	Chen Fang
**206 (2)**	**Optical properties of Weyl semimetals**
	Joel E. Moore
**208 (2)**	**3D quantum Hall effect**
	Hai-Zhou Lu
**210 (2)**	**Magnetic skyrmions: intriguing physics and new spintronic device concepts**
	Yan Zhou

### REVIEW

**Table:** 

**213 (2)**	**Topological quantum states of matter in iron-based superconductors: from concept to material realization**
	Ning Hao and Jiangping Hu

### INTERVIEW

**Table:** 

**227 (2)**	**Making the world from topological order**
	Philip Ball

## Genome Editing Research in China

### GUEST EDITORIAL

**Table:** 

**389 (3)**	**Preface to the special topic on genome editing research in China**
	Jinsong Li and Caixia Gao

### RESEARCH HIGHLIGHTS

**Table:** 

**391 (3)**	**Modulating gene translational control through genome editing**
	Feng Zhang and Daniel F. Voytas
**392 (3)**	**Genetic monkeys reveal a new role for a longevity protein in embryonic development**
	Yuyu Niu
**393 (3)**	**God does not play dice, and neither does CRISPR/Cas9**
	Chengzu Long

### PERSPECTIVES

**Table:** 

**394 (3)**	**Genome tagging project: tag every protein in mice through ‘artificial spermatids’**
	Jing Jiang, Meng Yan, Dangsheng Li and Jinsong Li
**397 (3)**	**Interrogating the noncoding genome in a high-throughput fashion**
	Zhuo Zhou and Wensheng Wei
**399 (3)**	**Genome editing in insects: current status and challenges**
	Jun Xu, Xia Xu, Shuai Zhan and Yongping Huang

### REVIEWS

**Table:** 

**402 (3)**	**Genome editing in large animals: current status and future prospects**
	Jianguo Zhao, Liangxue Lai, Weizhi Ji and Qi Zhou
**421 (3)**	**Gene editing in plants: progress and challenges**
	Yanfei Mao, Jose Ramon Botella, Yaoguang Liu and Jian-Kang Zhu
**438 (3)**	**Recent advances in structural studies of the CRISPR-Cas-mediated genome editing tools**
	Yuwei Zhu and Zhiwei Huang

### INTERVIEW

**Table:** 

**452 (3)**	**An exciting time for genome editing: an interview with David R. Liu**
	Weijie Zhao and Kevin T. Zhao

## Multiferroic Physics and Materials

### GUEST EDITORIAL

**Table:** 

**620 (4)**	**Multiferroics: a beautiful but challenging multi-polar world**
	Ce-Wen Nan and Jun-Ming Liu

### PERSPECTIVES

**Table:** 

**621 (4)**	**Perspective: voltage control of magnetization in multiferroic heterostructures**
	Jia-Mian Hu, Ce-Wen Nan and Long-Qing Chen
**624 (4)**	**Topological domains/domain walls and broken symmetries in multiferroics**
	Sang-Wook Cheong
**626 (4)**	**Multiferroics under the tip: probing magnetoelectric coupling at the nanoscale**
	Yunya Liu, Jan Seidel and Jiangyu Li

### REVIEWS

**Table:** 

**629 (4)**	**Magnetoelectricity in multiferroics: a theoretical perspective**
	Shuai Dong, Hongjun Xiang and Elbio Dagotto
**642 (4)**	**Lattice and spin dynamics in multiferroic BiFeO_3_ and *R*MnO_3_**
	Yan Song, Ben Xu and Ce-Wen Nan
**653 (4)**	**Single-phase multiferroics: new materials, phenomena, and physics**
	Chengliang Lu, Menghao Wu, Lin Lin and Jun-Ming Liu
**669 (4)**	**Structures and electronic properties of domain walls in BiFeO_3_ thin films**
	Huaixun Huyan, Linze Li, Christopher Addiego, Wenpei Gao and Xiaoqing Pan
**684 (4)**	**Topological domain states and magnetoelectric properties in multiferroic nanostructures**
	Guo Tian, Wenda Yang, Deyang Chen, Zhen Fan, Zhipeng Hou, Marin Alexe and Xingsen Gao

### INTERVIEW

**Table:** 

**703 (4)**	*** Pas de deux* of electricity and magnetism: an interview with Sang-Wook Cheong**
	Weijie Zhao

## The South China Sea Ocean Drilling

### GUEST EDITORIAL

**Table:** 

**870 (5)**	**New insights into marine basin opening**
	Pinxian Wang

### PERSPECTIVES

**Table:** 

**871 (5)**	**The role of magmatism in the thinning and breakup of the South China Sea continental margin**
	Zhen Sun, Jian Lin, Ning Qiu, Zhimin Jian, Pinxian Wang, Xiong Pang, Jinyun Zheng and Benduo Zhu
**877 (5)**	**Mantle upwelling beneath the South China Sea and links to surrounding subduction systems**
	Jian Lin, Yigang Xu, Zhen Sun and Zhiyuan Zhou
**881 (5)**	**Discovery of the marine Eocene in the northern South China Sea**
	Zhimin Jian, Haiyan Jin, Michael A. Kaminski, Fabricio Ferreira, Baohua Li and Pai-Sen Yu
**886 (5)**	**Intermingled fates of the South China Sea and Philippine Sea plate**
	Minghui Zhao, Jean-Claude Sibuet and Jonny Wu

### RESEARCH ARTICLE

**Table:** 

**891 (5)**	**Potential role of strike-slip faults in opening up the South China Sea**
	Chi-Yue Huang, Pinxian Wang, Mengming Yu, Chen-Feng You, Char-Shine Liu, Xixi Zhao, Lei Shao, Guangfa Zhong and Graciano P. Yumul Jr

### REVIEW

**Table:** 

**902 (5)**	**The South China Sea is not a mini-Atlantic: plate-edge rifting *vs* intra-plate rifting**
	Pinxian Wang, Chi-Yue Huang, Jian Lin, Zhimin Jian, Zhen Sun and Minghui Zhao

## Chemistry and Molecular Medicine

### GUEST EDITORIAL

**Table:** 

**1102 (6)**	**Molecular science *vs*. molecular medicine**
	Weihong Tan, Jianhui Jiang and Chaoyong Yang

### RESEARCH HIGHLIGHT

**Table:** 

**1103 (6)**	**Exosomal PD-L1: an effective liquid biopsy target to predict immunotherapy response**
	Yanling Song, Lingling Wu and Chaoyong Yang

### PERSPECTIVES

**Table:** 

**1105 (6)**	**Opportunities and challenges in the nanoparticles for nucleic acid therapeutics: the first approval of an RNAi nanoparticle for treatment of a rare disease**
	Xiawei Wei and Yuquan Wei
**1107 (6)**	**Precise design of nanomedicines: perspectives for cancer treatment**
	Jing Wang, Yiye Li, Guangjun Nie and Yuliang Zhao

### REVIEWS

**Table:** 

**1111 (6)**	**Phenotype and target-based chemical biology investigations in cancers**
	Guo-Qiang Chen, Ying Xu, Shao-Ming Shen and Jian Zhang
**1128 (6)**	**Supramolecular nanoscale drug-delivery system with ordered structure**
	Xin Jin, Lijuan Zhu, Bai Xue, Xinyuan Zhu and Deyue Yan
**1138 (6)**	**Influence of the microbiota on epigenetics in colorectal cancer**
	Danfeng Sun, Yingxuan Chen and Jing-Yuan Fang
**1149 (6)**	**Sin1-mediated mTOR signaling in cell growth, metabolism and immune response**
	Chun Ruan, Xinxing Ouyang, Hongzhi Liu, Song Li, Jingsi Jin, Weiyi Tang, Yu Xia and Bing Su

### INTERVIEW

**Table:** 

**1163 (6)**	**An interview with Feng Shao and Weihong Tan: chemistry intertwines with biology and medicine**
	Weijie Zhao

## EDITORIALS

**Table:** 

**1 (1)**	**China's reform of the regulatory system for medical products and its impact**
	Ruilin Song, Guowei Sang, Meiyu Geng and Hualiang Jiang
**183 (2)**	**Chinese science education in schools and beyond**
	Zhonghe Zhou
**377 (3)**	**Invitation to an open debate on non-human primates for research purposes**
**607 (4)**	**Culture and history of Chinese science**
	Mu-ming Poo
**855 (5)**	**Long march for healthy research ecology**
	Wei Yang
**1063 (6)**	**International exchange and collaboration in science**
	Mu-ming Poo

## FORUMS

**Table:** 

**171 (1)**	**Paleontology: advancing China's international leadership**
	Hepeng Jia
**369 (2)**	**Blockchain technology: development and prospects**
	Weijie Zhao
**595 (3)**	**Agriculture: science and technology safeguard sustainability**
	Hepeng Jia
**1054 (5)**	**A forum on innovative fusion approaches: will there be a SpaceX for fusion energy?**
	Weijie Zhao

## INTERVIEWS

**Table:** 

**177 (1)**	**Funding system reform for excellence in science: an interview with Jinghai Li, President of NSFC**
	Zhonghe Zhou and Weijie Zhao
**374 (2)**	**Jian-Wei Pan: building the quantum internet**
	Philip Ball
**601 (3)**	**The past and future of USTC: an interview with USTC President Xinhe Bao**
	Weijie Zhao and Xiaosu Yi
**839 (4)**	**Be a force for science: an interview with Barbara Schaal and Bill Moran**
	Jane Qiu
**1059 (5)**	**What will robots be like in the future?**
	Yanfeng Lu and Weijie Zhao
**1274 (6)**	**China's lunar and deep space exploration: touching the moon and exploring the universe**
	Weijie Zhao and Chi Wang

## FEATURED INSTITUTION

**Table:** 

**843 (4)**	**Dalian Institute of Chemical Physics, Chinese Academy of Sciences**
	Yan Zhang and Weijie Zhao

## COMMENTARIES

**Table:** 

**109 (1)**	**Editor's notes**
	Chung-I Wu
**123 (1)**	**Structural variation provides novel insights into dog domestication**
	Fuwen Wei
**123 (1)**	**Toward the identification and role of structural variations during dog domestication**
	Christophe Hitte
**124 (1)**	**Mining the hidden treasures from canid genomes**
	Fangqing Zhao
**289 (2)**	**Allo-parapatric speciation goes offshore**
	Trevor Price
**289 (2)**	**A new model of speciation**
	Loren H. Rieseberg
**290 (2)**	**A mixing-isolation-mixing model of speciation can potentially explain hotspots of species diversity**
	Richard J. Abbott
**291 (2)**	**Is speciation driven by cycles of mixing and isolation?**
	Nicholas H. Barton
**301 (2)**	**Mother tongue? Muttersprache? Lingua mater? Not so fast!**
	Dan Graur
**302 (2)**	**Macaque monkeys as a non-human primate circadian model**
	Han Wang
**494 (3)**	**After many a summer dies the swan**
	Chung-I Wu
**1004 (5)**	**Backward, upside down and inside out—the regulatory evolution of vertebrates**
	Chung-I Wu and Anlong Xu
**1014 (5)**	**Introduction of barley to the Tibetan Plateau: an important step toward Tibetan civilization**
	Song Ge
**1189 (6)**	**Mathematics of microRNAs: stabilizing gene regulatory networks**
	Victor Ambros

## CRITIQUES & DEBATES

### Geosciences

**Table:** 

**1094 (6)**	**A refutation of reported Levallois technology from Guanyindong Cave in south China**
	Feng Li, Yinghua Li, Xing Gao, Steven L. Kuhn, Eric Boëda and John W. Olsen
**1096 (6)**	**Robust technological readings identify integrated structures typical of the Levallois concept in Guanyindong Cave, south China**
	Yue Hu, Ben Marwick, Jia-Fu Zhang, Xue Rui, Ya-Mei Hou, Wen-Rong Chen, Wei-Wen Huang and Bo Li

## RESEARCH HIGHLIGHTS

### Biology & Biochemistry

**Table:** 

**859 (5)**	**From structure to function: unveiling the structure of the Na_v_ channel–toxin complex**
	Zhuan Zhou, Zuying Chai and Changhe Wang

### Chemistry

**Table:** 

**3 (1)**	**A head-to-toe makeover for classical sequencing-by-synthesis helps users to squeeze more out of each base**
	Jianbin Wang and Angela Wu
**609 (4)**	**Computationally designed protein activation**
	David Baker

### Computer Science

**Table:** 

**186 (2)**	**Deep Forest as a framework for a new class of machine-learning models**
	Lev V. Utkin, Anna A. Meldo and Andrei V. Konstantinov

### Engineering

**Table:** 

**185 (2)**	**New approach for efficient condensation heat transfer**
	Yaqi Cheng and Zuankai Wang

### Environment/Ecology

**Table:** 

**858 (5)**	**A question of balance: weighing the options for controlling ammonia, sulfur dioxide and nitrogen oxides**
	A.R. Ravishankara

### Materials Science

**Table:** 

**184 (2)**	**Wood-like polymeric materials by ice templating**
	Sylvain Deville
**380 (3)**	**Thermal conductivity of complex materials**
	G. Jeffrey Snyder, Matthias T. Agne and Ramya Gurunathan
**857 (5)**	**Stabilization of metastable structures from high pressure or high temperature to ambient conditions**
	Zujin Shi
**1065 (6)**	**Evaporation-assisted patterning beyond random assembly**
	Chuanhua Duan
**1066 (6)**	**Going slippery for a robust triboelectric nanogenerator**
	Wenluan Zhang, Qiangqiang Sun and Xu Deng
**1068 (6)**	**The rise of 3D cellular spheroids: efficient culture via upward growth from a superamphiphobic surface**
	Paul J. Dyson

### Mathematics

**Table:** 

**1064 (6)**	**Network science meets algebraic topology**
	Lingqing Shen, David M. Walker and Michael Small

### Physics

**Table:** 

**2 (1)**	**The race for quantum supremacy: pushing the classical limit for photonic hardware**
	Nicolò Spagnolo and Fabio Sciarrino
**378 (3)**	**Quantum oscillations turn log (*B*)-periodic in Dirac semimetals: ‘Who ordered that?’**
	Ziqiang Wang
**379 (3)**	**Dancing of electromagnetic spectra driven by metasurfaces**
	Min Gu
**608 (4)**	**A classical algorithm to mimic quantum decisions**
	Mile Gu
**856 (5)**	**Measuring the Meissner effect at megabar pressures**
	Dmitrii Semenok and Artem R. Oganov
	**Editors' selection of papers from China's academic journals [5(1), 188(2)]**

## PERSPECTIVES

### Biology & Biochemistry

**Table:** 

**611 (4)**	**Dog10K: the International Consortium of Canine Genome Sequencing**
	Guo-Dong Wang, Greger Larson, Jeffrey M. Kidd, Bridgett M. vonHoldt, Elaine A. Ostrander and Ya-Ping Zhang
**861 (5)**	**Ultrastructural cellular signatures: does cellular form follow function?**
	Manfred Auer
**864 (5)**	**Single-particle cryo-EM: beyond the resolution**
	Jean-Paul Armache and Yifan Cheng

### Chemistry

**Table:** 

**8 (1)**	**The 18-electron rule for main-group alkaline earth octacarbonyl complexes**
	Xueming Yang
**191 (2)**	**Condensed-matter chemistry: from materials to living organisms**
	Ruren Xu, Kui Wang, Gang Chen and Wenfu Yan

### Computer Science

**Table:** 

**616 (4)**	**A perspective on Gaussian processes for Earth observation**
	Gustau Camps-Valls, Dino Sejdinovic, Jakob Runge and Markus Reichstein

### Environment/Ecology

**Table:** 

**386 (3)**	**Unravelling the complexity in achieving the 17 sustainable-development goals**
	Bojie Fu, Shuai Wang, Junze Zhang, Zengqian Hou and Jinghai Li
**1076 (6)**	**Regional scientific research benefits threatened-species conservation**
	Yisi Hu, Zhenhua Luo, Colin A. Chapman, Stuart L. Pimm, Samuel T. Turvey, Michael J. Lawes, Carlos A. Peres, Tien Ming Lee and Pengfei Fan
**1080 (6)**	**Three global conditions for biodiversity conservation and sustainable use: an implementation framework**
	Harvey Locke, Erle C. Ellis, Oscar Venter, Richard Schuster, Keping Ma, Xiaoli Shen, Stephen Woodley, Naomi Kingston, Nina Bhola, Bernardo B. N. Strassburg, Axel Paulsch, Brooke Williams and James E. M. Watson

### Geosciences

**Table:** 

**10 (1)**	**Natatanuran frogs used the Indian Plate to step-stone disperse and radiate across the Indian Ocean**
	Zhi-Yong Yuan, Bao-Lin Zhang, Christopher J. Raxworthy, David W. Weisrock, Paul M. Hime, Jie-Qiong Jin, Emily M. Lemmon, Alan R. Lemmon, Sean D. Holland, Michelle L. Kortyna, Wei-Wei Zhou, Min-Sheng Peng, Jing Che and Elizabeth Prendini
**14 (1)**	**A framework for the regional critical zone classification: the case of the Chinese Loess Plateau**
	Yihe Lü, Jian Hu, Bojie Fu, Paul Harris, Lianhai Wu, Xiaolin Tong, Yingfei Bai and Alexis J. Comber
**384 (3)**	**Fire records in glacier ice**
	Chao You and Tandong Yao
**614 (4)**	**Intercalibration of ^40^Ar/^39^Ar laboratories in China, the USA and Russia for Emeishan volcanism and the Guadalupian–Lopingian boundary**
	Brian R. Jicha, Brad S. Singer and Youjuan Li
**1082 (6)**	**Deep neural network for remote-sensing image interpretation: status and perspectives**
	Jiayi Li, Xin Huang and Jianya Gong
**1086 (6)**	***Dacrycarpus* pattern shedding new light on the early floristic exchange between Asia and Australia**
	Xinkai Wu, Xiaoyan Liu, Tatiana Kodrul, Cheng Quan and Jianhua Jin

### Molecular Biology & Genetics

**Table:** 

**867 (5)**	**eGPS 1.0: comprehensive software for multi-omic and evolutionary analyses**
	Dalang Yu, Lili Dong, Fangqi Yan, Hailong Mu, Bixia Tang, Xiao Yang, Tao Zeng, Qing Zhou, Feng Gao, Zhonghuang Wang, Ziqian Hao, Hongen Kang, Yi Zheng, Hongwei Huang, Yuzhang Wei, Wei Pan, Yaochen Xu, Junwei Zhu, Shilei Zhao, Ciran Wang, Pengyu Wang, Long Dai, Mushan Li, Li Lan, Yiwei Wang, Hua Chen, Yi-Xue Li, Yun-Xin Fu, Zhen Shao, Yiming Bao, Fangqing Zhao, Luo-Nan Chen, Guo-Qing Zhang, Wenming Zhao and Haipeng Li

### Multidisciplinary

**Table:** 

**1091 (6)**	**Paradigm shift in science with tackling global challenges**
	Jinghai Li and Wenlai Huang

### Plant & Animal Science

**Table:** 

**1073 (6)**	**ATP translocation and chloroplast biology**
	Chia P. Voon and Boon L. Lim

### Physics

**Table:** 

**382 (3)**	**The frontier and perspective for tokamak development**
	Jiangang Li, Mingjiu Ni and Yu Lu
**1070 (6)**	**On the periodic solutions of the three-body problem**
	Shijun Liao and Xiaoming Li
**1071 (6)**	**Two-dimensional phonon engineering triggers microscale thermal functionalities**
	Run Hu and Xiaobing Luo

## MEETING NEWS

**Table:** 

**19 (1)**	**Challenges and opportunities of software automation discussed at the Yanqi-Lake Meeting**
	He Jiang
**619 (4)**	**International collaboration strives for resilient Silk Road at SiDRR Conference 2019**
	Rongzhi Tan and Peng Cui
**1100 (6)**	**A meeting to celebrate the centennial birthday of Yuan-Cheng Fung: the father of modern biomechanics and foreign member of the Chinese Academy of Sciences**
	Yi Cao

## RESEARCH ARTICLES

### Biology & Biochemistry

**Table:** 

**257 (2)**	***Complement C7* is a novel risk gene for Alzheimer's disease in Han Chinese**
	Deng-Feng Zhang, Yu Fan, Min Xu, Guihong Wang, Dong Wang, Jin Li, Li-Li Kong, Hejiang Zhou, Rongcan Luo, Rui Bi, Yong Wu, Guo-Dong Li, Alzheimer's Disease Neuroimaging Initiative (ADNI), Ming Li, Xiong-Jian Luo, Hong-Yan Jiang, Liwen Tan, Chunjiu Zhong, Yiru Fang, Chen Zhang, Nengyin Sheng, Tianzi Jiang and Yong-Gang Yao
**293 (2)**	**Reconciling the father tongue and mother tongue hypotheses in Indo-European populations**
	Menghan Zhang, Hong-Xiang Zheng, Shi Yan and Li Jin
**1191 (6)**	**Molecular game theory for a toxin-dominant food chain model**
	Bowen Li, Jonathan R. Silva, Xiancui Lu, Lei Luo, Yunfei Wang, Lizhen Xu, Aerziguli Aierken, Zhanserik Shynykul, Peter Muiruri Kamau, Anna Luo, Jian Yang, Deyuan Su, Fan Yang, Jianmin Cui, Shilong Yang and Ren Lai

### Chemistry

**Table:** 

**730 (4)**	**Electrochemical synthesis of nitric acid from air and ammonia through waste utilization**
	Yuting Wang, Yifu Yu, Ranran Jia, Chao Zhang and Bin Zhang
**929 (5)**	**Immobilization of functional nano-objects in living engineered bacterial biofilms for catalytic applications**
	Xinyu Wang, Jiahua Pu, Yi Liu, Fang Ba, Mengkui Cui, Ke Li, Yu Xie, Yan Nie, Qixi Mi, Tao Li, Lingli Liu, Manzhou Zhu and Chao Zhong
**1169 (6)**	**Visually constructing the chemical structure of a single molecule by scanning Raman picoscopy**
	Yao Zhang, Ben Yang, Atif Ghafoor, Yang Zhang, Yu-Fan Zhang, Rui-Pu Wang, Jin-Long Yang, Yi Luo, Zhen-Chao Dong and J. G. Hou

### Environment/Ecology

**Table:** 

**505 (3)**	**Altered trends in carbon uptake in China's terrestrial ecosystems under the enhanced summer monsoon and warming hiatus**
	Honglin He, Shaoqiang Wang, Li Zhang, Junbang Wang, Xiaoli Ren, Lei Zhou, Shilong Piao, Hao Yan, Weimin Ju, Fengxue Gu, Shiyong Yu, Yuanhe Yang, Miaomiao Wang, Zhongen Niu, Rong Ge, Huimin Yan, Mei Huang, Guoyi Zhou, Yongfei Bai, Zongqiang Xie, Zhiyao Tang, Bingfang Wu, Leiming Zhang, Nianpeng He, Qiufeng Wang and Guirui Yu
**515 (3)**	**An observation-based perspective of winter haze days in four major polluted regions of China**
	Lu Mao, Run Liu, Wenhui Liao, Xuemei Wang, Min Shao, Shaw Chen Liu and Yuanhang Zhang
**746 (4)**	**Climate and litter C/N ratio constrain soil organic carbon accumulation**
	Guoyi Zhou, Shan Xu, Philippe Ciais, Stefano Manzoni, Jingyun Fang, Guirui Yu, Xuli Tang, Ping Zhou, Wantong Wang, Junhua Yan, Gengxu Wang, Keping Ma, Shenggong Li, Sheng Du, Shijie Han, Youxin Ma, Deqiang Zhang, Juxiu Liu, Shizhong Liu, Guowei Chu, Qianmei Zhang, Yuelin Li, Wenjuan Huang, Hai Ren, Xiankai Lu and Xiuzhi Chen

### Engineering

**Table:** 

**1266 (6)**	**Ionic thermal up-diffusion in nanofluidic salinity-gradient energy harvesting**
	Rui Long, Zhengfei Kuang, Zhichun Liu and Wei Liu

### Geosciences

**Table:** 

**495 (3)**	**Uplift, climate and biotic changes at the Eocene–Oligocene transition in south-eastern Tibet**
	Tao Su, Robert A. Spicer, Shi-Hu Li, He Xu, Jian Huang, Sarah Sherlock, Yong-Jiang Huang, Shu-Feng Li, Li Wang, Lin-Bo Jia, Wei-Yu-Dong Deng, Jia Liu, Cheng-Long Deng, Shi-Tao Zhang, Paul J. Valdes and Zhe-Kun Zhou
**786 (4)**	**Exposures to temperature beyond threshold disproportionately reduce vegetation growth in the northern hemisphere**
	Xiuchen Wu, Weichao Guo, Hongyan Liu, Xiaoyan Li, Changhui Peng, Craig D. Allen, Cicheng Zhang, Pei Wang, Tingting Pei, Yujun Ma, Yuhong Tian, Zhaoliang Song, Wenquan Zhu, Yang Wang, Zongshan Li and Deliang Chen
**1016 (5)**	**Evidence for diurnal periodicity of earthquakes from midnight to daybreak**
	Jinlai Hao, Jinhai Zhang and Zhenxing Yao
**1024 (5)**	**Tianshanbeilu and the Isotopic Millet Road: reviewing the late Neolithic/Bronze Age radiation of human millet consumption from north China to Europe**
	Tingting Wang, Dong Wei, Xien Chang, Zhiyong Yu, Xinyu Zhang, Changsui Wang, Yaowu Hu and Benjamin T. Fuller
**1239 (6)**	**Solving the mystery of vanishing rivers in China**
	Yichu Wang, Jinren Ni, Yao Yue, Jiaye Li, Alistair G. L. Borthwick, Ximing Cai, An Xue, Li Li and Guangqian Wang
**1247 (6)**	**Isotopic evidence for volatile replenishment of the Moon during the Late Accretion**
	Yanhao Lin and Wim van Westrenen

### Information Science

**Table:** 

**74 (1)**	**Deep forest**
	Zhi-Hua Zhou and Ji Feng
**775 (4)**	**Detection for disease tipping points by landscape dynamic network biomarkers**
	Xiaoping Liu, Xiao Chang, Siyang Leng, Hui Tang, Kazuyuki Aihara and Luonan Chen
**962 (5)**	**Totally homogeneous networks**
	Dinghua Shi, Linyuan Lü and Guanrong Chen
**970 (5)**	**Bio-inspired untethered fully soft robots in liquid actuated by induced energy gradients**
	Liang Xiong Lyu, Fen Li, Kang Wu, Pan Deng, Seung Hee Jeong, Zhigang Wu and Han Ding

### Materials Science

**Table:** 

**64 (1)**	**A general aerosol-assisted biosynthesis of functional bulk nanocomposites**
	Qing-Fang Guan, Zi-Meng Han, Tong-Tong Luo, Huai-Bin Yang, Hai-Wei Liang, Si-Ming Chen, Guang-Sheng Wang and Shu-Hong Yu
**239 (2)**	**Li-ion battery material under high pressure: amorphization and enhanced conductivity of Li_4_Ti_5_O_12_**
	Yanwei Huang, Yu He, Howard Sheng, Xia Lu, Haini Dong, Sudeshna Samanta, Hongliang Dong, Xifeng Li, Duck Young Kim, Ho-kwang Mao, Yuzi Liu, Heping Li, Hong Li and Lin Wang
**247 (2)**	**Bio-inspired low-tortuosity carbon host for high-performance lithium-metal anode**
	Yi-Chen Yin, Zhi-Long Yu, Zhi-Yuan Ma, Tian-Wen Zhang, Yu-Yang Lu, Tao Ma, Fei Zhou, Hong-Bin Yao and Shu-Hong Yu
**532 (3)**	**Twist-driven separation of *p*-type and *n*-type dopants in single-crystalline nanowires**
	Dong-Bo Zhang, Xing-Ju Zhao, Gotthard Seifert, Kinfai Tse and Junyi Zhu
**540 (3)**	**SLIPS-TENG: robust triboelectric nanogenerator with optical and charge transparency using a slippery interface**
	Wanghuai Xu, Xiaofeng Zhou, Chonglei Hao, Huanxi Zheng, Yuan Liu, Xiantong Yan, Zhengbao Yang, Michael Leung, Xiao Cheng Zeng, Ronald X. Xu and Zuankai Wang
**758 (4)**	**Material patterning on substrates by manipulation of fluidic behavior**
	Yitan Li, Hao Wang, Henglu Xu, Shiting Wu, Xuemei Li, Jiapeng Yu, Chaoyu Huang, Zeyao Zhang, Hao Sun, Lu Han, Meihui Li, Anyuan Cao, Zhenhai Pan and Yan Li
**767 (4)**	**Rock-salt and helix structures of silver iodides under ambient conditions**
	Hongyang Huang, Jinying Zhang, Yifan Zhang, Chengcheng Fu, Jialiang Huang, Yonghong Cheng, Chunming Niu, Xinluo Zhao and Hisanori Shinohara
**944 (5)**	**Superior performance and high service stability for GeTe-based thermoelectric compounds**
	Tong Xing, Qingfeng Song, Pengfei Qiu, Qihao Zhang, Xugui Xia, Jincheng Liao, Ruiheng Liu, Hui Huang, Jiong Yang, Shengqiang Bai, Dudi Ren, Xun Shi and Lidong Chen
**955 (5)**	**Aging amorphous/crystalline heterophase PdCu nanosheets for catalytic reactions**
	Hongfei Cheng, Nailiang Yang, Xiaozhi Liu, Qinbai Yun, Min Hao Goh, Bo Chen, Xiaoying Qi, Qipeng Lu, Xiaoping Chen, Wen Liu, Lin Gu and Hua Zhang
**1255 (6)**	**Durable superamphiphobic silica aerogel surfaces for the culture of 3D cellular spheroids**
	Lianyi Xu, Shuangshuang Chen, Xuemin Lu and Qinghua Lu

### Molecular Biology & Genetics

**Table:** 

**110 (1)**	**Structural variation during dog domestication: insights from gray wolf and dhole genomes**
	Guo-Dong Wang, Xiu-Juan Shao, Bing Bai, Junlong Wang, Xiaobo Wang, Xue Cao, Yan-Hu Liu, Xuan Wang, Ting-Ting Yin, Shao-Jie Zhang, Yan Lu, Zechong Wang, Lu Wang, Wenming Zhao, Bing Zhang, Jue Ruan and Ya-Ping Zhang
**275 (2)**	**Speciation with gene flow via cycles of isolation and migration: insights from multiple mangrove taxa**
	Ziwen He, Xinnian Li, Ming Yang, Xinfeng Wang, Cairong Zhong, Norman C. Duke, Chung-I Wu and Suhua Shi
**455 (3)**	**DAZL is a master translational regulator of murine spermatogenesis**
	Haixin Li, Zhuqing Liang, Jian Yang, Dan Wang, Hanben Wang, Mengyi Zhu, Baobao Geng and Eugene Yujun Xu
**469 (3)**	**Ecological principle meets cancer treatment: treating children with acute myeloid leukemia with low-dose chemotherapy**
	Yixin Hu, Aili Chen, Xinchang Zheng, Jun Lu, Hailong He, Jin Yang, Ya Zhang, Pinpin Sui, Jingyi Yang, Fuhong He, Yi Wang, Peifang Xiao, Xin Liu, Yinmei Zhou, Deqing Pei, Cheng Cheng, Raul C. Ribeiro, Shaoyan Hu and Qian-fei Wang
**480 (3)**	**Transgenic rhesus monkeys carrying the human *MCPH1* gene copies show human-like neoteny of brain development**
	Lei Shi, Xin Luo, Jin Jiang, Yongchang Chen, Cirong Liu, Ting Hu, Min Li, Qiang Lin, Yanjiao Li, Jun Huang, Hong Wang, Yuyu Niu, Yundi Shi, Martin Styner, Jianhong Wang, Yi Lu, Xuejin Sun, Hualin Yu, Weizhi Ji and Bing Su
**739 (4)**	**Asymmetric biotic interchange across the Bering land bridge between Eurasia and North America**
	Dechun Jiang, Sebastian Klaus, Ya-Ping Zhang, David M. Hillis and Jia-Tang Li
**993 (5)**	**Evolutionary transition between invertebrates and vertebrates via methylation reprogramming in embryogenesis**
	Xiaocui Xu, Guoqiang Li, Congru Li, Jing Zhang, Qiang Wang, David K. Simmons, Xuepeng Chen, Naveen Wijesena, Wei Zhu, Zhanyang Wang, Zhenhua Wang, Bao Ju, Weimin Ci, Xuemei Lu, Daqi Yu, Qian-fei Wang, Neelakanteswar Aluru, Paola Oliveri, Yong E. Zhang, Mark Q. Martindale and Jiang Liu
**1005 (5)**	**Neolithic millet farmers contributed to the permanent settlement of the Tibetan Plateau by adopting barley agriculture**
	Yu-Chun Li, Jiao-Yang Tian, Feng-Wen Liu, Bin-Yu Yang, Kang-Shu-Yun Gu, Zia Ur Rahman, Li-Qin Yang, Fa-Hu Chen, Guang-Hui Dong and Qing-Peng Kong
**1176 (6)**	**Gene regulatory network stabilized by pervasive weak repressions: microRNA functions revealed by the May–Wigner theory**
	Yuxin Chen, Yang Shen, Pei Lin, Ding Tong, Yixin Zhao, Stefano Allesina, Xu Shen and Chung-I Wu
**1201 (6)**	**Prioritizing natural-selection signals from the deep-sequencing genomic data suggests multi- variant adaptation in Tibetan highlanders**
	Lian Deng, Chao Zhang, Kai Yuan, Yang Gao, Yuwen Pan, Xueling Ge, Yaoxi He, Yuan Yuan, Yan Lu, Xiaoxi Zhang, Hao Chen, Haiyi Lou, Xiaoji Wang, Dongsheng Lu, Jiaojiao Liu, Lei Tian, Qidi Feng, Asifullah Khan, Yajun Yang, Zi-Bing Jin, Jian Yang, Fan Lu, Jia Qu, Longli Kang, Bing Su and Shuhua Xu

### Multidisciplinary

**Table:** 

**55 (1)**	**Mapping intrinsic electromechanical responses at the nanoscale via sequential excitation scanning probe microscopy empowered by deep data**
	Boyuan Huang, Ehsan Nasr Esfahani and Jiangyu Li

### Neuroscience

**Table:** 

**87 (1)**	**BMAL1 knockout macaque monkeys display reduced sleep and psychiatric disorders**
	Peiyuan Qiu, Jian Jiang, Zhen Liu, Yijun Cai, Tao Huang, Yan Wang, Qiming Liu, Yanhong Nie, Fang Liu, Jiumu Cheng, Qing Li, Yun-Chi Tang, Mu-ming Poo, Qiang Sun and Hung-Chun Chang
**101 (1)**	**Cloning of a gene-edited macaque monkey by somatic cell nuclear transfer**
	Zhen Liu, Yijun Cai, Zhaodi Liao, Yuting Xu, Yan Wang, Zhanyang Wang, Xiaoyu Jiang, Yuzhuo Li, Yong Lu, Yanhong Nie, Xiaotong Zhang, Chunyang Li, Xinyan Bian, Mu-ming Poo, Hung-Chun Chang and Qiang Sun
**982 (5)**	**Scalable volumetric imaging for ultrahigh-speed brain mapping at synaptic resolution**
	Hao Wang, Qingyuan Zhu, Lufeng Ding, Yan Shen, Chao-Yu Yang, Fang Xu, Chang Shu, Yujie Guo, Zhiwei Xiong, Qinghong Shan, Fan Jia, Peng Su, Qian-Ru Yang, Bing Li, Yuxiao Cheng, Xiaobin He, Xi Chen, Feng Wu, Jiang-Ning Zhou, Fuqiang Xu, Hua Han, Pak-Ming Lau and Guo-Qiang Bi
**1223 (6)**	**High-resolution mapping of brain vasculature and its impairment in the hippocampus of Alzheimer's disease mice**
	Xiaochuan Zhang, Xianzhen Yin, Jingjing Zhang, Anan Li, Hui Gong, Qingming Luo, Haiyan Zhang, Zhaobing Gao and Hualiang Jiang

### Physics

**Table:** 

**231 (2)**	**Programmable time-domain digital-coding metasurface for non-linear harmonic manipulation and new wireless communication systems**
	Jie Zhao, Xi Yang, Jun Yan Dai, Qiang Cheng, Xiang Li, Ning Hua Qi, Jun Chen Ke, Guo Dong Bai, Shuo Liu, Shi Jin, Andrea Alù and Tie Jun Cui
**524 (3)**	**Stoichiometric evolutions of PH_3_ under high pressure: implication for high-*T*_c_ superconducting hydrides**
	Ye Yuan, Yinwei Li, Guoyong Fang, Guangtao Liu, Cuiying Pei, Xin Li, Haiyan Zheng, Ke Yang and Lin Wang
**707 (4)**	**Observation of acoustic spin**
	Chengzhi Shi, Rongkuo Zhao, Yang Long, Sui Yang, Yuan Wang, Hong Chen, Jie Ren and Xiang Zhang
**713 (4)**	**High-temperature superconductivity in sulfur hydride evidenced by alternating-current magnetic susceptibility**
	Xiaoli Huang, Xin Wang, Defang Duan, Bertil Sundqvist, Xin Li, Yanping Huang, Hongyu Yu, Fangfei Li, Qiang Zhou, Bingbing Liu and Tian Cui
**719 (4)**	**Universal bound on sampling bosons in linear optics and its computational implications**
	Man-Hong Yung, Xun Gao and Joonsuk Huh
**914 (5)**	**Log-periodic quantum magneto-oscillations and discrete-scale invariance in topological material HfTe_5_**
	Huichao Wang, Yanzhao Liu, Yongjie Liu, Chuanying Xi, Junfeng Wang, Jun Liu, Yong Wang, Liang Li, Shu Ping Lau, Mingliang Tian, Jiaqiang Yan, David Mandrus, Ji-Yan Dai, Haiwen Liu, Xincheng Xie and Jian Wang
**921 (5)**	**Physical modeling and validation of porpoises' directional emission via hybrid metamaterials**
	Erqian Dong, Yu Zhang, Zhongchang Song, Tianye Zhang, Chen Cai and Nicholas X. Fang

## REVIEWS

### Engineering

**Table:** 

**324 (2)**	**Nanomechanics of graphene**
	Yujie Wei and Ronggui Yang

### Environment/Ecology

**Table:** 

**349 (2)**	**When multi-functional landscape meets critical zone science: advancing multi-disciplinary research for sustainable human well-being**
	Ying Luo, Yihe Lü, Bojie Fu, Paul Harris, Lianhai Wu and Alexis Comber
**825 (4)**	***Ulva prolifera* green-tide outbreaks and their environmental impact in the Yellow Sea, China**
	Yongyu Zhang, Peimin He, Hongmei Li, Gang Li, Jihua Liu, Fanglue Jiao, Jianheng Zhang, Yuanzi Huo, Xiaoyong Shi, Rongguo Su, Naihao Ye, Dongyan Liu, Rencheng Yu, Zongling Wang, Mingjiang Zhou and Nianzhi Jiao

### Geosciences

**Table:** 

**125 (1)**	**Water in the upper mantle and deep crust of eastern China: concentration, distribution and implications**
	Qun-Ke Xia, Jia Liu, István Kovàcs, Yan-Tao Hao, Pei Li, Xiao-Zhi Yang, Huan Chen and Ying-Ming Sheng
**359 (2)**	**The 600-mm precipitation isoline distinguishes tree-ring-width responses to climate in China**
	Yu Liu, Huiming Song, Changfeng Sun, Yi Song, Qiufang Cai, Ruoshi Liu, Ying Lei and Qiang Li
**579 (3)**	**Exploring atmospheric free-radical chemistry in China: the self-cleansing capacity and the formation of secondary air pollution**
	Keding Lu, Song Guo, Zhaofeng Tan, Haichao Wang, Dongjie Shang, Yuhan Liu, Xin Li, Zhijun Wu, Min Hu and Yuanhang Zhang
**796 (4)**	**Linking atmospheric pollution to cryospheric change in the Third Pole region: current progress and future prospects**
	Shichang Kang, Qianggong Zhang, Yun Qian, Zhenming Ji, Chaoliu Li, Zhiyuan Cong, Yulan Zhang, Junming Guo, Wentao Du, Jie Huang, Qinglong You, Arnico K. Panday, Maheswar Rupakheti, Deliang Chen, Örjan Gustafsson, Mark H. Thiemens and Dahe Qin
**1040 (5)**	**Applications of chemical imaging techniques in paleontology**
	Yanhong Pan, Liang Hu and Tao Zhao

### Materials Science

**Table:** 

**304 (2)**	**Flow units as dynamic defects in metallic glassy materials**
	Zheng Wang and Wei-Hua Wang
**551 (3)**	**Cell membrane-covered nanoparticles as biomaterials**
	Mingjun Xuan, Jingxin Shao and Junbai Li
**562 (3)**	**The revival of thermal utilization from the Sun: interfacial solar vapor generation**
	Lin Zhou, Xiuqiang Li, George W. Ni, Shining Zhu and Jia Zhu

### Molecular Biology & Genetics

**Table:** 

**810 (4)**	**Dog10K: an international sequencing effort to advance studies of canine domestication, phenotypes and health**
	Elaine A. Ostrander, Guo-Dong Wang, Greger Larson, Bridgett M. vonHoldt, Brian W. Davis, Vidhya Jagannathan, Christophe Hitte, Robert K. Wayne, Ya-Ping Zhang and the Dog10K Consortium

### Physics

**Table:** 

**145 (1)**	**Probing primordial gravitational waves: Ali CMB Polarization Telescope**
	Hong Li, Si-Yu Li, Yang Liu, Yong-Ping Li, Yifu Cai, Mingzhe Li, Gong-Bo Zhao, Cong-Zhan Liu, Zheng-Wei Li, He Xu, Di Wu, Yong-Jie Zhang, Zu-Hui Fan, Yong-Qiang Yao, Chao-Lin Kuo, Fang-Jun Lu and Xinmin Zhang
**155 (1)**	**Recent progress in the study of transition in the hypersonic boundary layer**
	Cunbiao Lee and Shiyi Chen

